# Mediterranean Diet and Physical Exercise for Cardiometabolic Health: A Narrative Review

**DOI:** 10.3390/nu18152548

**Published:** 2026-08-04

**Authors:** Mohammad Aminullah Nurain Binti, Mónika Fekete, János Tamás Varga

**Affiliations:** 1 Department of Pulmonology, Semmelweis University, 1083 Budapest, Hungary; nurain.mohammad@stud.semmelweis.hu; 2 Institute of Preventive Medicine and Public Health, Semmelweis University, 1089 Budapest, Hungary; fekete.monika@semmelweis.hu

**Keywords:** Mediterranean diet, physical activity, exercise, cardiometabolic health, cardiovascular disease, lifestyle intervention, narrative review

## Abstract

Background: Cardiovascular disease (CVD) remains a leading cause of morbidity and mortality worldwide. The Mediterranean diet (MD) and regular physical activity are established components of cardiovascular prevention, but evidence regarding their combined cardiometabolic effects remains heterogeneous. Objective: This narrative review critically summarizes current evidence on the joint role of the MD and physical exercise in cardiometabolic health. Methods: A structured literature search was conducted in PubMed, Web of Science, and Scopus using terms related to the MD, CVD, cardiometabolic health, physical activity, and exercise. Experimental and observational human studies published between 2016 and 2026 were considered, together with relevant systematic reviews and meta-analyses. Particular attention was given to 10 key human studies, including randomized intervention studies and observational cohorts. Owing to substantial heterogeneity in populations, interventions, comparators, follow-up periods, and outcomes, the evidence was synthesized narratively. Results: The key studies generally reported favorable changes in body weight and composition, blood pressure, glucose regulation, and lipid profiles. One observational study also found a lower likelihood of cardiovascular medication initiation among participants with sustained adherence to both lifestyle components. However, most outcomes were surrogate cardiometabolic markers, and the independent effects of diet and exercise were often difficult to isolate. Conclusions: The MD and regular physical activity appear to be complementary components of cardiometabolic prevention. However, current evidence does not establish a consistent additive or synergistic effect. Further long-term, well-controlled studies are needed to clarify their independent and combined contributions across different populations.

## 1. Introduction

Globally, CVD is a major cause of death and disability, accounting for approximately 17.8 million deaths in 2017, or 31.8% of all deaths worldwide. The global burden of CVD continues to rise, with 612 million prevalent cases reported in 2021 [1,2]. Although age-standardized mortality and disability-adjusted life years have declined over recent decades, population aging and the increasing prevalence of modifiable cardiometabolic risk factors continue to drive the absolute burden of disease [1,2]. Obesity, hypertension, diabetes, dyslipidemia, physical inactivity, and unhealthy dietary patterns are among the principal modifiable determinants of CVD, collectively highlighting the central role of lifestyle-based prevention [3,4,5].

Current cardiovascular prevention guidelines increasingly emphasize dietary patterns rather than isolated nutrients or foods. Among these, the Mediterranean diet (MD)—characterized by a high intake of vegetables, fruits, legumes, nuts, whole grains, and olive oil; moderate consumption of fish and fermented dairy products; and limited intake of red and processed meat—has been consistently associated with favorable cardiometabolic outcomes [6,7,8]. Evidence from epidemiological studies, randomized controlled trials, systematic reviews, and meta-analyses indicates that greater adherence to the MD is associated with lower cardiovascular risk and improvements in blood pressure, lipid profiles, glycemic regulation, body weight, and inflammatory status [9,10]. Many of these effects are attributed to the combined contribution of unsaturated fatty acids, dietary fiber, polyphenols, micronutrients, and other bioactive compounds rather than to any single dietary component. Increased consumption of plant-based foods, including vegetables, fruits, nuts, legumes, and whole grains, has also been associated with lower coronary heart disease risk and more favorable cardiometabolic profiles [11,12].

Regular physical activity represents a second major pillar of cardiovascular prevention. Aerobic, resistance, and combined exercise can improve blood pressure, insulin sensitivity, lipid metabolism, cardiorespiratory fitness, body composition, vascular function, and functional capacity. These benefits extend beyond direct cardiovascular adaptations and include reductions in visceral adiposity, systemic inflammation, and metabolic dysfunction. The available studies, however, differ substantially in exercise modality, intensity, frequency, supervision, and duration, contributing to variability in the observed outcomes [5,9,11,13,14,15,16].

The MD and physical exercise may influence several shared biological pathways, including energy balance, insulin signaling, endothelial function, nitric oxide bioavailability, lipid metabolism, oxidative stress, inflammation, and gut microbiome-related metabolic processes [17,18]. Their concurrent use is therefore biologically plausible as a complementary lifestyle strategy. Nevertheless, the available evidence does not consistently distinguish the independent effects of diet and exercise from those of energy restriction, weight-loss counseling, behavioral support, or other components of multimodal lifestyle interventions. Claims of additive or synergistic benefit should be interpreted cautiously [19,20].

Previous systematic reviews and meta-analyses have established the cardiovascular and metabolic benefits of the Mediterranean diet and physical activity, although these lifestyle components have often been evaluated separately or in relation to selected outcomes [9,21,22,23]. A previous systematic review and meta-analysis specifically examining their concurrent promotion reported favorable effects on body weight, body mass index, waist circumference, blood pressure, glucose regulation, insulin resistance, triglycerides, and cholesterol profiles. However, that analysis was limited to 11 randomized controlled trials published up to March 2018 and identified substantial between-study heterogeneity [23].

Since then, additional randomized and observational studies have expanded the evidence across diverse populations, exercise modalities, intervention designs, and cardiometabolic outcomes. Nevertheless, uncertainty remains regarding the independent, additive, or interactive contributions of diet and exercise because many interventions also incorporate energy restriction, behavioral counseling, weight-loss support, or other lifestyle components. Moreover, much of the available evidence concerns surrogate cardiometabolic risk markers—such as body weight, waist circumference, blood pressure, glucose, and lipid concentrations—rather than direct measures of cardiovascular function or major cardiovascular events.

Accordingly, this narrative review aims to critically synthesize current evidence on the combined role of the Mediterranean diet and physical exercise in cardiometabolic health. It distinguishes between clinical cardiovascular outcomes, surrogate cardiometabolic risk markers, and proposed biological mechanisms; examines heterogeneity across populations and intervention characteristics; and evaluates whether the available evidence supports complementary, potentially additive, or biologically plausible effects.

## 2. Methods

### 2.1. Review Design

This narrative review provides a structured synthesis of evidence on the combined role of the Mediterranean diet and physical exercise in cardiometabolic health. It integrates human intervention and observational studies, systematic reviews, meta-analyses, and selected mechanistic publications, while distinguishing clinical cardiovascular outcomes, surrogate cardiometabolic markers, and proposed biological mechanisms.

### 2.2. Structured Literature Search

PubMed/MEDLINE, Web of Science, and Scopus were searched for publications issued between January 2016 and June 2026. Earlier landmark studies and relevant reviews were also consulted to provide historical, clinical, and mechanistic context.

The search combined controlled vocabulary, where available, with free-text terms, including “Mediterranean diet”, “Mediterranean dietary pattern”, “physical activity”, “physical exercise”, “aerobic exercise”, “resistance exercise”, “combined exercise”, “cardiovascular disease”, “cardiometabolic health”, “cardiovascular risk”, “blood pressure”, “lipid profile”, “glucose metabolism”, “body composition”, “vascular function”, and “cardiorespiratory fitness”. Terms were combined using AND and OR. No language restrictions were applied. Reference lists of relevant reviews and key primary studies were also examined to identify additional publications relevant to the narrative synthesis.

### 2.3. Evidence Selection and Scope

Priority was given to human studies evaluating Mediterranean dietary patterns together with structured exercise, physical activity promotion, leisure-time physical activity, or habitual physical activity. Randomized and non-randomized intervention studies, cohort studies, systematic reviews, and meta-analyses were considered. Mechanistic studies were used selectively to support biological interpretation.

Relevant publications addressed at least one of the following domains:

1. Clinical cardiovascular outcomes, including cardiovascular events, mortality, medication initiation, and healthcare-related endpoints;
2. Surrogate cardiometabolic markers, including adiposity, blood pressure, glucose regulation, insulin sensitivity, and lipid profiles;
3. Functional or physiological outcomes, including cardiorespiratory fitness, vascular function, exercise capacity, and functional performance;
4. Biological pathways involving endothelial function, nitric oxide bioavailability, oxidative stress, inflammation, insulin signaling, lipid metabolism, body composition, or the gut microbiome.

Studies unrelated to the Mediterranean diet or cardiometabolic health were not prioritized. Animal and in vitro studies were not used as primary clinical evidence. Ten key human studies, comprising six randomized controlled trials and four cohort studies, were selected for detailed presentation because they directly examined Mediterranean dietary patterns combined with physical activity or structured exercise. They were treated as illustrative core evidence rather than as an exhaustive set of formally included studies.

### 2.4. Data Charting

For the key human studies, data were charted on publication year, country, study design, sample size, participant characteristics, baseline health status, dietary intervention, exercise modality, frequency, intensity, duration, supervision, adherence, comparator, follow-up, outcomes, and main findings.

Charted outcomes included anthropometric measures, blood pressure, glucose regulation, lipid profiles, inflammatory markers, cardiovascular medication use, vascular measures, and cardiorespiratory fitness. Data were checked for completeness and consistency before synthesis.

### 2.5. Critical Appraisal of the Key Human Evidence

Randomized controlled trials were appraised using the Cochrane Risk of Bias 2 tool across five domains: randomization, deviations from intended interventions, missing outcome data, outcome measurement, and selective reporting. Cohort studies were assessed using the Newcastle–Ottawa Scale, covering selection, comparability, and outcome ascertainment.

Two reviewers assessed study quality independently, with disagreements resolved by consensus. Appraisal results informed the interpretation of the evidence but were not used as formal eligibility criteria. Particular attention was given to adherence, self-reported exposures, residual confounding, incomplete data, selective reporting, short follow-up, and the absence of comparator groups capable of separating the effects of diet and exercise.

### 2.6. Narrative Synthesis

A narrative synthesis was used because of heterogeneity in participant characteristics, baseline risk, dietary prescriptions, energy restriction, exercise modality and dose, supervision, follow-up, adherence, comparators, and outcomes.

Evidence was organized by population, study design, intervention type, and outcome domain. Clinical cardiovascular outcomes were considered separately from surrogate markers and mechanistic evidence. Greater interpretive weight was given to randomized trials, prospective cohorts, systematic reviews, and meta-analyses.

Because few studies directly compared diet alone, exercise alone, and their combination, synergistic or superior effects were not assumed. Findings were interpreted as complementary, potentially additive, or biologically plausible, while accounting for additional intervention components such as energy restriction, behavioral counseling, and weight-loss support.

## 3. Results of the Evidence Synthesis

### 3.1. Methodological Quality of the Key Human Studies

**Figure 1:**
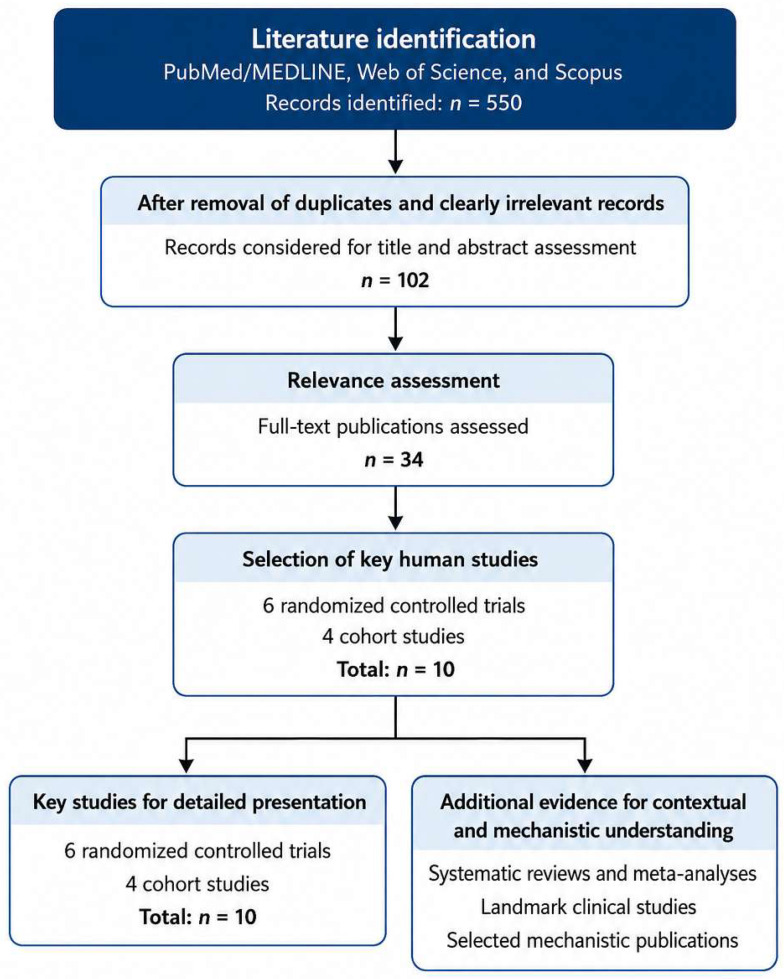
Structured literature search and selection of key evidence for the narrative review.

The literature identification process is summarized in Figure 1. The flow chart illustrates the structured search and selection of key evidence but does not represent a formal PRISMA-based study-selection procedure.

**Figure 2:**
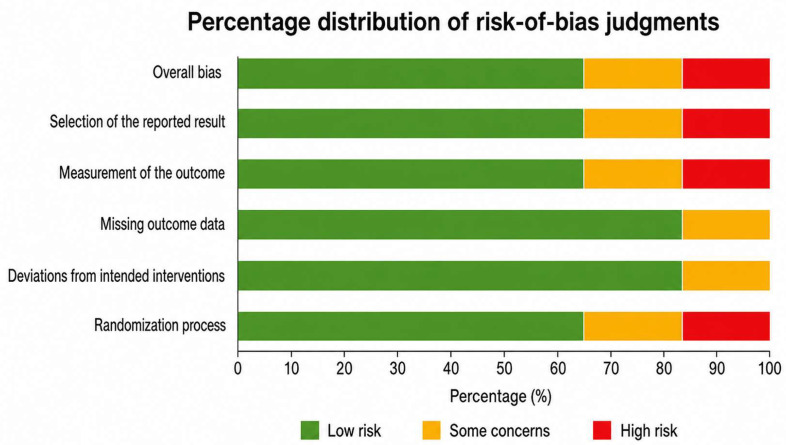
Risk-of-bias assessment of the key randomized controlled trials using the Cochrane RoB 2 tool.

The RoB 2 assessment of the randomized controlled trials is presented in Figure 2. One trial was rated as having a high overall risk of bias because of concerns regarding outcome measurement and selection of the reported results. Another trial showed a high risk of bias in the randomization domain. The remaining trials were rated as having either a low risk of bias or some concerns across the assessed domains. Two reviewers performed the assessments independently, and disagreements were resolved by consensus. The findings were used to inform the interpretation of the evidence rather than to determine study eligibility.

Four observational studies were assessed using the Newcastle–Ottawa Scale (Table 1). Scores ranged from 8 to 9 out of 9, indicating high methodological quality. All studies scored highly in the selection and outcome domains. Two studies received one of two possible stars for comparability because adjustment was limited to one major confounding factor. Residual confounding nevertheless remains possible because of the observational study designs.

### 3.2. Characteristics of the Key Human Studies

Ten key human studies were examined in detail because they evaluated Mediterranean dietary patterns together with structured exercise, physical activity promotion, or habitual physical activity. The studies were conducted in Croatia, Spain, Italy, Portugal, and Saudi Arabia and included adults with obesity, metabolic syndrome, elevated cardiovascular risk, or physical inactivity.

Sample sizes ranged from 52 to 7063 participants. The physical activity components included walking, leisure-time activity, aerobic exercise, resistance training, combined endurance and resistance exercise, and broader activity-promotion programmes. Several interventions also incorporated energy restriction, weight-loss counselling, or behavioral support.

Most outcomes were surrogate cardiometabolic markers, including body weight, waist circumference, visceral adiposity, blood pressure, glucose metabolism, lipid profiles, inflammatory indices, and body composition. One study assessed cardiovascular medication initiation. Direct cardiovascular events and direct measures of cardiovascular function were rarely reported. The study characteristics and main findings are summarized in Table 2.

Overall, the direction of the findings was generally favorable across several cardiometabolic domains, although the magnitude and consistency of the effects varied among studies (Table 2). However, the findings were not uniform, and most studies assessed surrogate risk markers rather than major cardiovascular events or direct measures of cardiovascular function. The studies also differed substantially in design, participant characteristics, intervention components, physical activity modalities, follow-up duration, and comparator conditions.

## 4. Mediterranean Diet and Cardiometabolic Risk

The Mediterranean diet reflects the traditional dietary pattern of countries surrounding the Mediterranean Sea and is characterized by a high intake of non-starchy vegetables, fruits, seeds, nuts, legumes, and minimally refined whole-grain cereals, with olive oil as the principal source of fat [17,33]. The MD is also regarded as an environmentally sustainable dietary pattern [34]. Epidemiological evidence has linked greater adherence to the MD with a lower prevalence of cardiovascular disease [35]. Meta-analyses of observational studies and clinical trials have further associated the MD with lower risks of cardiovascular disease, diabetes, myocardial infarction, coronary heart disease, neurodegenerative disorders, cancer, and all-cause mortality [36,37]. Its potential benefits have also been examined in relation to cognitive function, aging, and quality of life [38]. These broader effects have contributed to its inclusion in healthy-aging frameworks that emphasize the maintenance of physical, mental, and functional health and independence in daily activities [39,40].

Interest in Mediterranean dietary patterns was strongly influenced by the Seven Countries Study, which examined diet, lifestyle, cardiovascular risk factors, and mortality across contrasting populations. Men aged 40–59 years living in Mediterranean cohorts, including Dalmatia, Crevalcore, Montegiorgio, Crete, and Corfu, generally exhibited lower age-standardized rates of coronary heart disease mortality than participants in several northern and eastern European cohorts, particularly those in Finland and Slavonia [41].

The cardiometabolic effects of the MD are likely related to the combined contribution of its principal food groups and bioactive components rather than to a single nutrient. The main dietary components and their proposed cardiovascular relevance are summarized in Table 3.

## 5. Cardiometabolic Evidence for the Mediterranean Diet

### 5.1. Clinical Cardiovascular Outcomes

One of the largest prospective studies included 74,886 women from the Nurses’ Health Study who were followed for 20 years. Greater adherence to the Mediterranean diet, reflected by a higher Alternate Mediterranean Diet Score, was associated with a 29% lower risk of coronary heart disease (RR: 0.71; 95% CI: 0.62–0.82) and a non-significant 13% lower risk of stroke (RR: 0.87; 95% CI: 0.73–1.02) [47]. In the Health Professionals Follow-up Study and the Nurses’ Health Study, an increase in the Alternate Mediterranean Diet Score during the first four years of follow-up was associated with a 9% lower subsequent risk of cardiovascular disease (95% CI: 3–14%) over the following 20 years [48]. In the EPIC-Spain cohort, adherence to the MD was associated with a 27% lower incidence of CHD (RR: 0.73; 95% CI: 0.57–0.94) [49]. A two-point increase in the Mediterranean diet score was also associated with a 25% lower risk of all-cause mortality in a Greek population [50] and an 8% lower risk among older adults from nine European countries [51].

The Lyon Diet Heart Study provided important evidence for secondary cardiovascular prevention. This randomized controlled trial examined a Mediterranean-style diet enriched with alpha-linolenic acid in patients with a previous myocardial infarction over a follow-up period of approximately 46 months [52]. Participants assigned to the intervention showed a substantially lower risk of recurrent cardiovascular events, including cardiac death and nonfatal myocardial infarction, than those in the control group. In an observational analysis of participants in the GISSI-Prevenzione trial, greater adherence to dietary advice promoting the consumption of fish, fruits, olive oil, and raw and cooked vegetables was associated with an approximately 49% lower risk of all-cause mortality after myocardial infarction [53].

The PREDIMED trial was a landmark primary-prevention study involving 7447 participants at high cardiovascular risk. Participants were randomly assigned to a Mediterranean diet supplemented with extra-virgin olive oil, a Mediterranean diet supplemented with nuts, or a control diet based on advice to reduce dietary fat [54]. After a median follow-up of 4.8 years, the Mediterranean diet supplemented with extra-virgin olive oil was associated with a 31% lower risk of the composite endpoint of myocardial infarction, stroke, or cardiovascular death (HR: 0.69; 95% CI: 0.53–0.91), whereas the Mediterranean diet supplemented with nuts was associated with a 28% lower risk (HR: 0.72; 95% CI: 0.54–0.95), compared with the control diet. The trial was discontinued early following a prespecified interim analysis that demonstrated evidence of benefit in both Mediterranean diet groups [54].

A subsequent systematic review and meta-analysis including three randomized controlled trials and more than 9000 participants, including participants from the PREDIMED trial, reported a 38% lower incidence of cardiovascular disease with the Mediterranean diet compared with control diets (RR: 0.62; 95% CI: 0.50–0.78; two trials) and a 35% lower incidence of myocardial infarction (RR: 0.65; 95% CI: 0.49–0.88; two trials) [55]. These findings support a role for the Mediterranean diet in cardiovascular prevention, although the small number of randomized trials contributing to these estimates should be considered when interpreting the results.

### 5.2. Surrogate Cardiometabolic Risk Markers

Evidence regarding lipid outcomes is less consistent. A 2019 Cochrane systematic review and meta-analysis found that the Mediterranean diet produced a small reduction in total cholesterol of 0.16 mmol/L (95% CI: −0.32 to 0.00; five randomized controlled trials), with little or no effect on LDL cholesterol, HDL cholesterol, or triglycerides in some comparisons. In primary-prevention trials, the Mediterranean diet was associated with small reductions in LDL cholesterol of 0.15 mmol/L (95% CI: −0.27 to −0.02; seven trials) and triglycerides of 0.09 mmol/L (95% CI: −0.16 to −0.01; seven trials), but had little effect on total or HDL cholesterol [56].

Substudies of PREDIMED reported improvements in apolipoprotein B, apolipoprotein A-I, their ratio, LDL particle characteristics, and HDL function [57,58,59]. These observations are consistent with evidence concerning the effects of monounsaturated and polyunsaturated fatty acids on circulating lipids and lipoproteins [60]. A meta-analysis of randomized trials comparing red meat with other dietary sources found more favorable lipid changes when red meat was replaced by high-quality plant protein sources rather than fish or low-quality carbohydrates [61]. An umbrella review also associated the Mediterranean diet with improvements in body weight, body mass index, waist circumference, total and HDL cholesterol, C-reactive protein, and interleukin-6 compared with control diets [36].

Blood pressure findings have generally been favorable but modest. A meta-analysis of six trials involving more than 7000 participants found that adherence to the Mediterranean diet for at least one year reduced systolic blood pressure by approximately 1.44 mmHg and diastolic blood pressure by 0.70 mmHg, although the limited number of trials and substantial heterogeneity warrant cautious interpretation [62]. A separate meta-analysis of three randomized controlled trials reported reductions of 3.02 mmHg in systolic and 1.99 mmHg in diastolic blood pressure [63].

### 5.3. Vascular Function

Direct assessments of cardiovascular or vascular function have been less common than measurements of conventional risk markers. In an Australian randomized controlled intervention involving 166 adults older than 64 years, adherence to the Mediterranean diet was associated with modest reductions in systolic blood pressure after three and six months. At six months, flow-mediated dilation was 1.3 percentage points higher in the Mediterranean diet group than in the control group (95% CI: 0.2–2.4; *p* = 0.026), suggesting improved endothelial function [64]. 

This evidence indicates that the Mediterranean diet is associated with both clinical cardiovascular outcomes and favorable changes in surrogate cardiometabolic markers. However, these outcome categories should be interpreted separately, as changes in blood pressure, lipid concentrations, anthropometric measures, or vascular function do not directly demonstrate reductions in major cardiovascular events.

## 6. Physical Activity and Exercise in Cardiometabolic Prevention

Regular physical activity and reduced sedentary time are central components of cardiovascular prevention [65]. Promoting PA remains a major public health priority because physical inactivity contributes substantially to healthcare expenditure, chronic disease burden, premature mortality, and reduced life expectancy [66]. Regular PA is associated with lower risks of cardiovascular disease, type 2 diabetes, hypertension, obesity, and all-cause mortality [67]. Both aerobic and resistance exercise contribute to cardiovascular risk reduction, and clinically meaningful benefits may also occur at activity levels below current guideline recommendations [13,68]. Meeting recommended PA levels is generally associated with an approximately 20–30% lower risk of all-cause and cardiovascular mortality, although the magnitude of association varies according to population, activity measurement, intensity, and study design [69,70].

### 6.1. Recommendations for Physical Activity

Exercise prescription should be adapted to the individual’s clinical condition, functional capacity, and cardiovascular risk. For cardiovascular prevention, European guidelines recommend at least 150–300 min of moderate-intensity aerobic activity per week, 75–150 min of vigorous-intensity aerobic activity per week, or an equivalent combination of moderate- and vigorous-intensity activity [65,71,72]. 

Recommendations require additional individualization in patients with ischemic heart disease or heart failure. Although physical inactivity is an established risk factor for ischemic heart disease, vigorous exertion may transiently increase the risk of acute cardiovascular events in susceptible individuals. Exercise testing, and functional imaging when clinically indicated, may therefore be required to assess inducible myocardial ischemia, arrhythmias, abnormal hemodynamic responses, and an appropriate exercise intensity in patients with chronic coronary syndromes [73]. 

Exercise training is also recommended for patients with stable chronic heart failure [74]. The appropriate modality, intensity, and rate of progression should be individualized according to clinical stability, symptoms, functional capacity, comorbidities, and baseline exercise-test results. Aerobic exercise is generally appropriate for stable patients with New York Heart Association class I–III symptoms, whereas markedly deconditioned or more symptomatic patients should begin at a low intensity and progress gradually under appropriate clinical supervision [74,75].

### 6.2. Cardiovascular and Cardiometabolic Adaptations to Exercise

Exercise induces hemodynamic adaptations that increase oxygen delivery to working skeletal muscle and facilitate the removal of metabolic by-products. Acute responses include increased cardiac output through changes in heart rate, stroke volume, and myocardial contractility, together with redistribution of blood flow and a reduction in vascular resistance within active skeletal muscle [11,76,77].

Exercise modalities are commonly classified along a continuum from predominantly aerobic endurance activity to predominantly resistance or anaerobic exercise. Aerobic exercise produces sustained increases in cardiac output and stroke volume, whereas resistance exercise is generally characterized by more transient hemodynamic responses and greater increases in arterial pressure, depending on exercise intensity, muscle mass involved, and the use of the Valsalva maneuver [78,79].

Long-term endurance training may influence cardiac structure, including left ventricular chamber dimensions, wall thickness, and myocardial mass, although the magnitude of adaptation depends on training type, duration, sex, age, baseline fitness, and body size [80]. Endurance-trained athletes generally exhibit preserved systolic function and enhanced early diastolic filling, although resting left ventricular ejection fraction may be normal or mildly reduced because of increased ventricular dimensions and stroke volume [81].

### 6.3. Effects on Cardiometabolic Risk Markers

Much of the association between physical activity and lower cardiovascular disease risk may be mediated through changes in established cardiometabolic risk factors, including adiposity, blood pressure, glucose regulation, endothelial function, and lipid metabolism [5,13,16,82,83,84,85,86]. Exercise may lower blood pressure through reductions in sympathetic activity and vascular resistance, together with improvements in endothelial nitric oxide bioavailability and skeletal muscle microvascular perfusion. Its cardiovascular benefits are not explained solely by weight reduction, as favorable metabolic, cardiorespiratory, and vascular adaptations may occur independently of substantial weight loss. Exercise also improves insulin sensitivity, glucose disposal, and skeletal muscle glucose uptake, thereby contributing to diabetes prevention and glycemic control. Aerobic physical activity is generally associated with lower triglyceride and LDL cholesterol concentrations and higher HDL cholesterol, although the magnitude of these changes depends on exercise intensity, baseline lipid status, and training modality. These mechanisms provide biological support for the cardiometabolic benefits of physical activity, although their relative contributions vary according to exercise modality, intensity, duration, baseline health status, age, sex, and concurrent dietary or weight-loss interventions [5,13,16,82,83,84,85,86].

## 7. Discussion

This narrative review integrates evidence from 10 key human studies examining Mediterranean dietary patterns together with regular physical activity or structured exercise. Across these studies, the combined lifestyle approach was generally associated with improvements in anthropometric, glycemic, hemodynamic, and lipid-related outcomes. These findings are consistent with previous systematic reviews and meta-analyses showing that dietary modification and physical activity may exert complementary effects on cardiometabolic health through changes in metabolic regulation, endothelial function, inflammation, and body composition.

The evidence should, however, be interpreted in the context of substantial clinical and methodological heterogeneity. Study populations differed in age, baseline cardiovascular risk, body mass index, and clinical status, ranging from physically inactive adults without established disease to individuals with obesity, metabolic syndrome, or high cardiovascular risk (Table 2). Dietary approaches also varied, with some studies using energy-restricted Mediterranean diets and behavioral counselling, while others assessed habitual adherence in observational settings. Exercise interventions differed in modality, frequency, intensity, supervision, and duration and included walking, aerobic exercise, resistance training, and combined programmes. Follow-up ranged from six months to one year or longer, and outcomes included anthropometric measures, blood pressure, lipid and glycemic markers, inflammatory indices, medication use, and body composition. This heterogeneity limits direct comparison and prevents identification of an optimal dietary and exercise prescription [23,56].

The direction of the findings was generally favorable, but the available evidence relates mainly to surrogate cardiometabolic markers rather than direct cardiovascular function or major cardiovascular events [23]. The biological plausibility of complementary effects is supported by established mechanisms of both interventions [11,42,87]. The Mediterranean diet provides monounsaturated fatty acids, polyphenols, dietary fiber, antioxidants, and omega-3 fatty acids, which may support endothelial function and reduce oxidative stress and chronic low-grade inflammation [88]. Physical activity may complement these effects by improving insulin sensitivity, nitric oxide bioavailability, vascular function, autonomic regulation, and body composition [13]. These pathways may contribute to the observed changes in blood pressure, adiposity, dyslipidemia, and glucose metabolism, although most were not directly measured in the key human studies [11,42,87].

The findings are also consistent with landmark evidence on the Mediterranean diet alone. The PREDIMED trial reported an approximately 30% lower incidence of major cardiovascular events among participants assigned to Mediterranean diets supplemented with extra-virgin olive oil or nuts [54]. The Nurses’ Health Study and the Spanish EPIC cohort likewise associated greater adherence to Mediterranean dietary patterns with lower risks of coronary heart disease and cardiovascular mortality [47,89,90]. Evidence from randomized lifestyle trials suggests that interventions incorporating both Mediterranean dietary promotion and physical activity can improve several cardiometabolic risk markers [23]. However, the limited number of direct component-comparison studies prevents firm conclusions regarding whether the combined intervention provides benefits beyond those of either component alone [23].

Gut microbiota-related mechanisms may also contribute to the cardiometabolic effects of Mediterranean dietary patterns and physical activity [18,91,92,93]. Fiber- and polyphenol-rich foods can influence microbial composition and the production of short-chain fatty acids, while exercise has been associated with greater microbial diversity and improved microbial function [91]. These mechanisms remain biologically plausible but were not directly evaluated in most of the key studies and should therefore be regarded as hypotheses requiring targeted investigation.

Favorable associations were reported across older adults, individuals with obesity or metabolic syndrome, and participants at elevated cardiovascular risk. Sex-specific responses may also be relevant. Gorini et al. reported greater reductions in total and LDL cholesterol in men, whereas women showed greater improvements in HDL cholesterol [27]. These observations suggest that age, sex, baseline metabolic status, and exercise modality may influence the response to combined lifestyle interventions, although confirmation in adequately powered trials is needed.

The findings support current recommendations that place a healthy diet and regular physical activity at the center of cardiovascular prevention and management [65,71]. In clinical practice, Mediterranean dietary counselling and individualized exercise prescription may be considered complementary components of a comprehensive lifestyle programme [54]. Such approaches may improve several cardiovascular risk factors simultaneously, but they should not be presented as substitutes for evidence-based pharmacological treatment when medication is clinically indicated [71]. At the population level, improved access to healthy foods and opportunities for regular physical activity may help reduce the burden of cardiovascular disease and associated healthcare costs [54,65,71].

Several limitations should be acknowledged. In addition to the substantial clinical and methodological heterogeneity described above, dietary adherence and physical activity were frequently assessed by self-report, increasing the risk of recall and reporting bias. Several interventions had relatively short follow-up, preventing firm conclusions about long-term sustainability. In addition, many studies included energy restriction, behavioral support, or weight-loss counselling, making it difficult to isolate the independent effects of diet and exercise. Although the cohort studies generally showed high Newcastle–Ottawa Scale scores and most trials had acceptable methodological quality, differences in study design, intervention content, and outcome selection precluded quantitative pooling and required narrative synthesis [23].

Future studies should use clearer and more standardized definitions of Mediterranean dietary interventions, adherence measures, and behavioral support strategies [94]. Exercise protocols should report modality, intensity, frequency, duration, supervision, progression, tailoring, and adherence in accordance with established reporting recommendations [95]. Greater consistency is also needed in outcome selection, including blood pressure, lipid profile, glycemic control, inflammatory markers, vascular function, cardiorespiratory fitness, and major cardiovascular events. Adequately powered, long-term randomized controlled trials should directly compare diet alone, exercise alone, and their combination across different age groups, sexes, and cardiometabolic risk profiles. Such studies would clarify whether the effects are independent, additive, or interactive and would support translation into clinical practice and public health policy [96].

## 8. Conclusions

This narrative review indicates that Mediterranean dietary patterns combined with regular physical activity are associated with favorable changes in several cardiometabolic risk markers, including blood pressure, adiposity, glucose regulation, and lipid profiles. The available evidence is based mainly on surrogate outcomes, while direct evidence for major cardiovascular events and direct measures of cardiovascular function remains limited. The two lifestyle components are biologically complementary, but current studies do not establish a consistent additive or synergistic effect because diet, exercise, energy restriction, and behavioral support were often delivered together. Considerable heterogeneity in populations, interventions, follow-up, and outcomes also limits identification of an optimal combined strategy. 

Mediterranean dietary counselling and individualized physical activity can therefore be considered complementary elements of cardiovascular prevention. Further long-term, well-controlled trials are needed to determine the relative and combined contributions of these components and to identify the most effective approaches for different clinical and demographic groups.

## Figures and Tables

**Table 1:** Methodological quality of the key observational studies assessed using the Newcastle–Ottawa Scale.

Study	Selection (Max. 4 Stars)	Comparability (Max. 2 Stars)	Outcome (Max. 3 Stars)	Total Score (/9)	Quality
Di Renzo et al., 2020 [24]	★★★★	★	★★★	**8**	High
Ribó-Coll et al., 2021 [25]	★★★★	★★	★★★	**9**	High
Konieczna et al., 2023 [26]	★★★★	★★	★★★	**9**	High
Gorini et al., 2025 [27]	★★★★	★	★★★	**8**	High

Scoring interpretation based on the total Newcastle–Ottawa Scale score: high quality, 7–9 stars; moderate quality, 5–6 stars; and low quality, <5 stars. The maximum scores are four stars for Selection, two stars for Comparability, and three stars for Outcome, giving a maximum total score of nine stars.

**Table 2:** Characteristics and principal findings of key human studies evaluating Mediterranean dietary patterns in combination with physical activity or structured exercise.

First Author, Year	Country	Study Design	Participants	Intervention or Exposure	Main Cardiometabolic Findings
Pavić et al., 2019 [28]	Croatia	Single-blind RCT	Adults aged 18–69 years with obesity (BMI > 30 kg/m^2^)	Mediterranean diet combined with at least 30 min of group-based walking; 12-month intervention	Systolic and diastolic blood pressure decreased significantly from baseline (*p* = 0.001 and *p* = 0.037, respectively).
Salas-Salvadó et al., 2019 [29]	Spain	RCT	Adults aged 55–75 years with overweight or obesity and metabolic syndrome (*n* = 626)	Energy-restricted Mediterranean diet, physical activity promotion, and behavioral weight-loss support	Waist circumference, fasting glucose, triglycerides, and HDL cholesterol improved significantly compared with the control group (*p* < 0.002).
Fernández-García et al., 2020 [30]	Spain	RCT	Adults at high cardiovascular risk (*n* = 75; energy-restricted MD plus PA, *n* = 38; MD alone, *n* = 37)	Energy-restricted Mediterranean diet plus physical activity versus Mediterranean diet alone	No significant between-group differences were observed in serum polyamines or related metabolites.
Di Renzo et al., 2020 [24]	Italy	Observational cohort study	Women who completed the 6-month study (*n* = 52; mean age 47.3 ± 12.5 years)	Personalized Mediterranean diet combined with occupational, leisure-time, and organized physical activity	Several cardiovascular risk indices improved, whereas the neutrophil-to-lymphocyte and platelet-to-lymphocyte ratios remained unchanged.
Ribó-Coll et al. 2021 [25]	Spain	Cohort study	Adults at high cardiovascular risk (*n* = 7063); men aged 55–80 years and women aged 60–80 years	Mediterranean diet adherence and leisure-time physical activity	Sustained adherence to the Mediterranean diet and leisure-time physical activity was associated with a lower risk of initiating cardiovascular medication.
Muralidharan et al., 2021 [31]	Spain	Randomized lifestyle-intervention study	Men and women aged 55–75 years with overweight or obesity and metabolic syndrome	Energy-restricted Mediterranean diet and physical activity promotion	Body weight decreased by a median of 4.2 kg in the intervention group (change: −4.2 kg; IQR, −6.8 to −2.5), compared with 0.2 kg in the control group (change: −0.2 kg; IQR, −2.1 to 1.4; *p* < 0.001).
Konieczna et al., 2023 [26]	Spain	Cohort study (PREDIMED)	Older adults with overweight or obesity and metabolic syndrome (*n* = 1521; intervention, *n* = 760; control, *n* = 761)	Energy-restricted Mediterranean diet and physical activity promotion	The intervention reduced total and visceral adiposity and attenuated age-related losses of lean mass.
Barbosa et al., 2024 [21]	Portugal	RCT	Adults at high cardiovascular risk (*n* = 102; mean age 70.1 ± 7.9 years)	Mediterranean-inspired diet combined with physical exercise	Waist circumference (*p* = 0.002), bicipital skinfold thickness (*p* < 0.001), visceral fat (*p* < 0.001), and triglycerides (*p* = 0.029) decreased significantly compared with the control condition.
Prieto-González et al., 2025 [32]	Saudi Arabia	RCT	Physically inactive adults aged 35–50 years (*n* = 125; 61 men and 64 women), without cardiovascular, metabolic, or musculoskeletal disease	Mediterranean diet combined with three endurance and two resistance-training sessions per week	Body mass, BMI, body-fat percentage, waist circumference, waist-to-hip ratio, systolic and diastolic blood pressure, heart rate, double product, glucose, and LDL cholesterol decreased significantly from baseline (all *p* < 0.05).
Gorini et al., 2025 [27]	Italy	Observational cohort study	Adults with overweight or obesity (*n* = 205; 107 men and 98 women) who self-selected aerobic, anaerobic, combined, or no-activity groups	Mediterranean diet combined with different self-selected physical activity patterns	Men showed greater reductions in total and LDL cholesterol, whereas women showed an increase in HDL cholesterol. Fasting glucose decreased in both sexes. Anaerobic activity was associated with greater lipid improvements in men, while aerobic activity produced the most favorable metabolic changes in women.

Abbreviations: BMI, body mass index; BP, blood pressure; BST, bicipital skinfold thickness; CG, control group; CVD, cardiovascular disease; HDL, high-density lipoprotein; IG, intervention group; IQR, interquartile range; LDL, low-density lipoprotein; LTPA, leisure-time physical activity; MD, Mediterranean diet; PA, physical activity; RCT, randomized controlled trial; TG, triglycerides; WC, waist circumference; WHR, waist-to-hip ratio.

**Table 3:** Principal components of the Mediterranean diet and their proposed cardiovascular benefits.

Mediterranean Diet Component	Examples	Proposed Cardiovascular Benefits
Unsaturated fats [42]	Olive oil	Improved endothelial function and reduced systemic inflammation
Whole grains and dietary fiber [43]	Oats and barley	Modest favorable effects on total and LDL cholesterol and, in some populations, blood pressure
Nuts and legumes [44]	Walnuts, peanuts, almonds, and legumes	Reductions in total and LDL cholesterol and potential improvements in inflammatory and oxidative-stress markers
Fruits and vegetables [45]	Tomatoes, spinach, oranges, and berries	Increased antioxidant capacity and potential reductions in blood pressure
omega-3 PUFAs [46]	Fish	Triglyceride lowering and potential cardiovascular benefits through effects on inflammation, oxidation, endothelial function, and thrombosis; effects on arrhythmias and major cardiovascular outcomes remain heterogeneous

Abbreviations: LDL, low-density lipoprotein; omega-3 PUFAs, omega-3 polyunsaturated fatty acids.

## Data Availability

No new data were created or analyzed in this study. Data sharing is not applicable to this article.
